# The Relationship Between Social Media Digitalization and Coronavirus Disease 2019 Fear Among Service Sector Employees

**DOI:** 10.3389/fpsyg.2021.702423

**Published:** 2021-12-02

**Authors:** Kai Wang, Kejun Lin, Shixin Yang, Sang-Gyun Na

**Affiliations:** ^1^Business School, Gansu University of Political Science and Law, Lanzhou, China; ^2^Department of Business Administration, Wonkwang University, Iksan, South Korea

**Keywords:** COVID-19 vaccine, employee performance, social media, fear, China

## Abstract

In the age of digitalization, social media has played a significant role in quickly spreading the news about current affairs. From December 2019 to now, coronavirus disease 2019 (COVID-19), with its several mutated shapes, has more transmissible potential catastrophe and has become a severe phenomenon issue worldwide. The international spread of the epidemic has created fear among people, especially employees working physically in different organizations. The present research aimed to measure the impact of social media on its users in the China. The social media users more often were influenced by shocking news instructively and destructively. The research analysis was based on service sector employees and data collected from 630 respondents via a structured questionnaire. This research was confirmed the negative impact of fear on social media on the performance of employees. This research was also confirmed the moderation impact of the COVID-19 vaccine on the relationship between social media fear and employee performance. This research recommends that the China Censor Board checked the news and its validity to reduce the fear of COVID-19 among employees. This research will become a roadmap for organizations and media controllers to understand the impact of social media during an intense situation. The telecommunication sector will reduce psychological disease and enhance the work capability of employees by controlling unnecessary and unapproved material about sensitive issues.

## Introduction

The recent study initiatives, emphases of China media, and self-reporting social media are increasing fear and melancholy due to the outbreak of novel coronavirus disease 2019 (COVID-19) worldwide. Media plays an essential role in spreading awareness for some currently occurring issues, such as COVID-19, which is becoming the source of disease spread worldwide (Moghe et al., 2020). COVID-19 epidemic evolved from China, and after some days, this disease became severe grave in the social, economic, and health systems of the world. According to Kufel (2020), the role of social media on COVID-19 coverage is pretty unacceptable while talking about electronic media, such as television, that shows news about COVID-19. Still, news coverage and transmissions are without solid proof from medical professionals. Social media users frequently use famous social media applications, such as WeChat, Weibo, Toutiao, Facebook, YouTube, and WhatsApp. There are no special programs arranged to inform, encourage, or motivate the morale of the public and protect them from mental stress in this global crisis. Everyone has become an expert about COVID-19, only to comment on their public posts instead of using this media platform to help miserable people. Many social media users used this platform only to spread fake news about COVID-19. Still, at the same time, there is thinking that television transmissions regularly use alarming and shocking words. Social media news disseminates most of the talks about death and dissatisfactions about life and hopes, as it sometimes seems that there is a jungle of deaths and more and more deaths are coming soon. The media is spreading fear and stress instead of encouraging (Sarfraz et al., 2018; Rehman et al., 2021).

Providing accurate information and aiding knowledge among social media of citizens is called upon to take responsibility. Frenkel et al. (2020) indicated that once the WHO complained that social media organizations have been spreading false information over COVID-19 worldwide; few social media groups used their platforms to spread incorrect data and tried to remove it. A recent study described the effect of media on the health of people (Mohsin et al., 2020b; Salamat et al., 2020; Naseem et al., 2021; Sarfraz et al., 2021). Muwahed (2020) stated that social media had affected the shocking crisis over some countries while many people have been greedy for buying foodstuff and household things because of the widespread fear of COVID-19. Merchant and Lurie (2020) investigated that social media is responsible for panic and creates a fear of shortage of foodstuff in the surrounding. A universal situation is leading in which social media organizations have taken steps to remove fake posts about COVID-19. Besides, Victor (2020) reported that people saw empty store posts on social media, spreading fear of food shortages. In addition, Kelvin and Rubino (2020) noted that social media put everyone at risk of having a ratio of one to another. They heard surprising news about why people posted on social media as soon as possible.

Furthermore, it has been distinguished that the publication of misleading information on social media networks, where almost all media channels are exposed to diseases, has harmful effects on general public health and mental health (Frenkel et al., 2020). Victor (2020) claimed that this is an age of digital information today. Chinese population cannot get facts about COVID-19, and social media users share fake news, information, photos, and videos. Similarly, the Indian government said the highest social media application corporations, such as WeChat, Weibo, Toutiao, YouTube, Facebook, Tiktok, WhatsApp, and Twitter, to break in their journey of publishing fake information because it spread anxiety and fear to the public. This fake news highly impacted the younger generations that use social media. The 21st century has seen a change in the way that the public embraces online communication technology. The new upgrading media terms became an essential source of public discussion, non-experiments, criticisms, health, disease, and treatment (Muwahed, 2020; Rothschild, 2020).

In the same way, Wongkoblap et al. (2017) noted that human beings are spending a lot of time on social media and have seen many infected panic attacks in several countries during the COVID-19 epidemic, spreading anxiety. Similarly, Jee (2020) indicated that everyone is a pseudo-professional who tries to raise his or her voice and transfer messages to almost all COVID-19. Respectively, Ittefaq et al. (2020) explained that we empowered social media to create fear around COVID-19 because we all distributed anxiety, fear news, and socialized among the public. Because Coronavirus has spread worldwide, there is misinformation about it. There is no difference between social media corporations to stop its spread. WeChat, Weibo, Toutiao, Facebook, Google, and Twitter said that they were removing misinformation about the Coronavirus as quickly as possible. It worked with the WHO and other government organizations to ensure that human beings have found correct facts. However, New York Times brings out, for example, dozens of movies, photos, and written posts shared on every social media platform that is believed to be cracking (Depoux et al., 2020). These posts are no longer limited to English. Many languages have started from Hindi and Urdu to Hebrew and Persian, reflecting the virus’s spreading cycle as it has traveled worldwide (Dunnan et al., 2020). Security researchers also say that hackers set up worn-out websites that claimed to contain fake data on almost coronavirus. These sites were honestly virtual traps, intended to steal non-public information or break gadgets of people who came to them. Spreading false and malicious material about the Coronavirus has been a perfect reminder of the war of attrition fought by researchers and internet groups. Even if corporations have decided to protect this fact, they are frequently raided by liars and thieves on the internet. Ahmed et al. (2020) observed many ways to disseminate facts and information to the community today with the upgradation of social media.

Studies show that the fear of an illness and any resulting behavioral changes may be passed on through a virus (Asmundson and Taylor, 2020). There are historical instances of people fearing mass contagious illnesses based on media reports, such as when London had a massive plague in 1665. It was linked to stories in the newspapers, which forced officials to close the printing presses. Studies also have indicated that media coverage of past outbreaks of pandemic diseases has led to a rise in worry (Tausczik et al., 2012). Extensive research should be done on the mechanisms by which the media incite fear and other emotions in the face of a pandemic. Additional studies should be conducted to determine the specific factors that increase the likelihood of certain persons these symptoms. Emotion contagion is one area where a little study has been conducted.

This research aims to measure the impact of social media on the performance of employees of industries during COVID-19. This research also identifies the toxicities of speech freedom about the pandemic and curbing dissenting political views on social media. The commoner does not know about the facts and figures for a specific situation. When an individual speaks out in an extreme case, one presents their mental state and psychological effect on the reader. The pandemic phenomenon reflects health issues other than a pandemic, which reduces the performance levels of employees. This overreach is destined to evoke an intense reaction and severe to alienate the general public from its telecommunication approach at a time when a unified stance is essential.

## Literature Review

### Employee Performance

As a dependent variable in this study, the performance of the workers is taken into consideration. It is the performance of workers in an industry that industrialists are most concerned about since the profit or loss of a sector is directly linked to the performance of employees. The outbreak of COVID-19 had a significant impact on employee performance in a variety of ways, such as lockdown, frightening news about the pandemic, limited working locations, and stringent operating procedures, among others. The basic rationale for selecting this variable is real, as the success of the industries and the performance of the workers are very complementary to one another. Because the economy is in such bad shape, the luckiest individuals are included among those working (Jamil et al., 2021). The performance of workers increases the productivity of the industry and the achievement of goals and objectives on time. As a result of the epidemic, human resource managers were pressed to continue with the recruitment, selection processes, employee engagement, and training and development initiatives (Jamil et al., 2021). In this catastrophic global lockdown scenario, the oil industry is humiliated for the first time as a barrel of oil drops to less than zero for the first time in its history. The management of safety performance and the management of safety information are inextricably linked to one another in many ways (Kim et al., 2021). Physical workplaces have been transformed into virtual workplaces, suitable for the services industry but not the manufacturing sector. After using Partial Least Squares Structural Equation Modeling (PLS-SEM) and the Input-Processes-Outcomes (IPO) framework, Bartsch et al. (2020) discovered that service workers were able to live virtually with little difficulty. Contrary to expectations, individual job autonomy and team cohesion had little impact on the work performance of services workers; this was unexpected and work-related.

### COVID-19 Worry and Social Media Fear

In late December 2019, when China reported a mysterious illness in Wuhan city called COVID-19, some cases were registered due to this illness. The infection has spread around the world and has become a pandemic disease. The telecommunications institution plays an essential role in the awareness of people about the effects of the disease. Still, at the same time, media became a source of spreading fear and anxiety among their users. The social media platforms gave their users different instructions about preventing this virus, such as washing their hands, wearing the mask, and keeping distance among themselves (Ahmed et al., 2020; Lee, 2020), but at the same time, social media became a source of fear dissemination during COVID-19. According to Lee (2020), this news of COVID-19 became one of the deadliest public disastrous news in human history.

**FIGURE 1:**
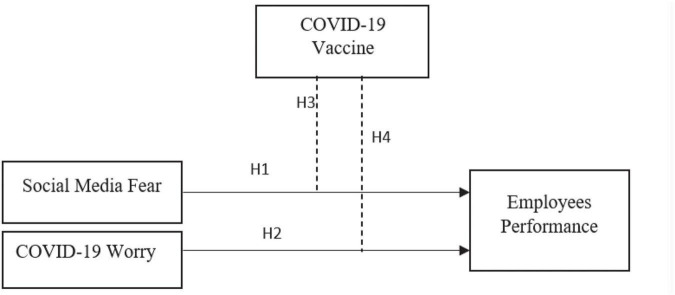
Theoretical framework.

All developed and developing countries trying to cope with the epidemic and save the health of human beings should take essential preventive measures, such as lockdown, contact tracing patients, green health code, and quarantine (Khan and Naushad, 2020). Until now, the impact of this virus has been severe in emerging countries, such as China. There was a lack of medical setup, public health literacy to respond to the crisis (Jamil et al., 2021). In addition, the outbreaks spreading misinformation from social media among the people create fear and anxiety. The misinformation calamity for emerging economies is also tricky due to the lack of health infrastructure, lack of deontological expertise in health hospitals, lack of trusty media, and health knowledge among the public. The WHO rules on pandemics help people avoid this sudden condition (Jee, 2020; Lee, 2020). In Figure 1, the relationship and direction are influencing the scaling of variables (Jamil et al., 2021). The information about the COVID-19 is spreading greater than the virus itself, leading to full-size public panic worldwide (Frenkel et al., 2020). Social media, alternatively, is a realistic platform for spreading public health messages to the audience Ida et al. (2020). Brewer has published on BBC News (Emmott, 2020) that the general public has been shocked and horrified to hear all kinds of information about COVID-19, causing them to land in a state of depression (Dunnan et al., 2020).

Additionally, Pennycook et al. (2020) claims that panic is spreading among social media users. Simultaneously, in a conversation on social media (Ida et al., 2020; Mohsin et al., 2020a), people almost rely on social media to get information and facts about COVID-19. Some countries use news filters that are why social media presents some points but not all correct records. After the emergence of COVID-19 and the migration of countries beyond mainland China, people turned to social media to get more information about the virus. In just 24 h, according to Pennycook et al. (2020), there were 19 million mentions of the COVID-19 cases on social media and news sites worldwide.

Similarly, Shimizu (2020) stated that many nations were no longer aware of the spread of COVID-19 or failed to provide the information they needed. Because of this, people trusted information they could find on social media. Khan and Naushad (2020) contend that a global changing condition impacts in disclosure of fake news and misinformation about COVID-19, which is alleged to be the “beginning” of a virus based on misinformation lab theory contained in social media (Jamil et al., 2021). Accordingly, Frenkel et al. (2020) are confident that “media reporting has highlighted COVID-19 as an utterly exclusive threat as opposed to people that have caused panic and created tension.” Victor (2020) described that social media plays three crucial roles in spreading information about COVID-19 in most countries (Jamil et al., 2021). Firstly, a factor about the occurrence of the virus was posted on social media. Secondly, facts about the epidemic appeared on social media as misinformation, fake news, and misleading. Thirdly, social media has caused widespread anxiety, fear, and depression worldwide during the outbreak of COVID-19.

It seems that pleasure, anger, and worry are all socially infectious types of emotion. Research has shown that when it comes to strangers, especially the spread of negative emotions (Paukert et al., 2008). Individuals who have greater levels of emotion contagion tend to respond more strongly to distressing experiences (Trautmann et al., 2018). That so, those who are sensitive to the emotions of others may be more vulnerable to the COVID-19 epidemic.

Even though most research on emotion contagion has focused on human interactions, a recent study has shown that digital communication may be a conduit for emotional contagion (Coviello et al., 2014; Goldenberg and Gross, 2020). The fact that social media is very popular in the contemporary world and that many people get their news from social media emphasizes the significance of this. Since it has spread worldwide, this sickness has gotten a great deal of attention in the media. Previous research has shown that more media exposure will result in increased anxiety about COVID-19; however, this increase among concerns may be much more severe in individuals who are very susceptible to emotional contagion. Hence, we suggest the following hypothesis:

H1: Social media fear significantly influences employee performance.
H2: COVID-19 worry significantly influences employee performance.

### Moderating Role of COVID-19 Vaccine

Workers are the foundation of any business, and the primary duty of any organization is to ensure that its workers are safe and secure, particularly during this difficult period of a pandemic outbreak. With the COVID-19 pandemic in full swing, it is clear that companies must take their responsibilities to safeguard workers seriously and that the widespread distribution of COVID-19 vaccinations is necessary to preserve their health even in the face of widespread vaccination reluctance. According to a recent study, businesses of all sizes must act responsibly to ensure that no harm is done, care for their employees, and allow the government to do its job in protecting people. Additionally, every company should make concentrated efforts to meet employee needs and desires cost-effectively to improve the performance of the company. Exposure of workers to COVID-19 may be reduced by providing safe and healthy working circumstances, such as appropriate protective equipment and training and personal hygiene practices, such as hand washing and coughing etiquettes. The first step is to gather current information about their situation (Jamil et al., 2021; Shah et al., 2021). A variety of approaches may be used to reduce direct human-to-human interaction throughout the job process. Ensure that employees are aware of the coronavirus and the immunization program by providing them with appropriate information distribution. Governments and corporate sectors must safeguard the health of their workers during this difficult period, but this crisis also presents a chance for our economy to reach a watershed moment.

The vaccine of COVID-19 is used as a moderator variable between the performance of the employee, COVID-19 worry, and social media. The vaccine is performing two rules at a time in this research. First, it will point out the psychological impact on the employees of industries who are afraid of COVID-19 due to media. Second, it will also measure the effectiveness of vaccines because the immune system of the human body is related to mental health. The accurate effect of the vaccine is not only dependent on the vaccine but also characterized the vaccinated person. The psychological factors, i.e., stress, depression, and loneliness, which are created by exaggerating spreading fear by media, may affect the immune system of respondents. The vaccine of COVID-19 is the best moderator variable that can elaborate the intensity of the relationship between the dependent and independent variables. The inflammatory markers of vaccines rise within hours due to Innate Immune Response in normal human beings, but its effect and reaction period are prolonged for stressed people. Again, the fake news of social media restricted the general public from accepting the vaccine of COVID-19. The useful information and encouraging vaccine material are rare on social media than negative and discoursing materials (Salali and Uysal, 2020; Madison et al., 2021; Murphy et al., 2021). Hence, we proposed the following hypothesis:

H3: COVID-19 vaccine moderates the relationship between social media fear and employee performance.
H4: COVID-19 vaccine moderates the relationship between COVID-19 fear and employee performance.

As per previous literature, most research is available on social media fear and COVID-19, but the performance of employees and vaccines are still undiscussed. In this section, the theoretical information is categorized for two purposes, i.e., an indication of previous research and direction and the current inclusion of this research work. The independent variable is explained in detail with cited references, but vaccine and employee performance have only declared the moderator and dependent variables due to unavailable literature.

## Methodology

This study designed a questionnaire according to the hypotheses stated above. The participants in this study were experienced users of social media platforms in China. A self-administered questionnaire was used to collect data from respondents. The research analysis was based on service sector employees, and data were collected from 630 respondents via a structured questionnaire. A pilot study with 100 participants was carried out. Since providing recommendations, revisions were made to the final questionnaire to make it more understandable for the respondents of the study. To ensure the content validity of the measures, three academic management experts analyzed and made improvements in the items of constructs. The experts searched for spelling errors, grammatical errors, and ensured that the items were correct. The experts have proposed minor revisions to social media fear and employee performance items and advised that the original number of items be maintained. This study used an online community to invite social media users to complete the designated online questionnaire system. Online questionnaires have the following advantages (Tan and Teo, 2000): (1) sampling is not restricted to a single geological location, (2) lower cost, and (3) faster questionnaire responses. A total of 650 questionnaires were returned from respondents. There were 630 appropriate replies considered for the final analysis. Data were analyzed through MS Excel and Statistical Package for the Social Sciences (SPSS) software.

### Measures

The study used items established from prior research to confirm the reliability and validity of the measures. All items are evaluated through five-point Likert-type scales where “1” denotes strongly disagree, “3” denotes neutral, and “5” denotes strongly agree. We used 10 items adopted from a prior study by Bartsch et al. (2020) to get responses about employee performance. COVID-19 worry and social media fear were measured with eight items adapted from the study of Pennycook et al. (2020). Finally, the COVID-19 vaccine was measured with seven items adapted from the prior study of Murphy et al. (2021).

## Results

### Demographic Characteristics of Respondents

This study analyzed the data through the SPSS. Primary data were collected from 630 respondents, and demographic characteristics of the work position of respondents, such as age and gender, are illustrated in Table 1.

**TABLE 1:** Profile of respondents.

**Range**	**Frequency**	**Percent**
**Gender**		
Male	190	56.7
Female	145	43.3
Total	335	100
**Age**		
21–30	149	44.4
31–40	103	30.7
41–50	80	23.8
51 years or above	3	0.89
**Work position**		
Executive	08	2.3
CEO	02	0.59
Manager	91	27.16
Senior manager	21	6.26
Office worker	69	20.59
Others	144	42.98

Data collection for this study was performed using a questionnaire. In this study, we analyzed the impact of social media fear on the performance of employees through the moderating role of the COVID-19 vaccine. Before testing the structural model, the measurement model was analyzed in terms of construct reliability, convergent validity, and discriminant validity. According to our assessment of the reliability of the indicators, there are 25 indicators with outer loading greater than 0.70 (Table 2). A total of 10 indicators of social media fear were used, all showing a reliable factor loading, as indicated in Table 3. The COVID-19 vaccine is measured through eight items which also show factor loading greater than 0.70. The employee performance is measured through seven items, and all the outer loading greater than seven is significantly reliable.

**TABLE 2:** Reliability and validity.

**Variables**	**Mean**	***SD***	**Cronbach’s Alpha**
Social media fear	3.690	0.341	0.745
COVID-19 worry	3.145	0.390	0.890
Employees performance	3.765	0.390	0.821
COVID-19 vaccine	3.431	0.545	0.789

**TABLE 3:** Direct and indirect effects.

	**Coeff.**	***SD***	***T*-values**	***P* values**
CVD VC – > EP	0.054	0.031	3.540	0.000
SMF – > EP	0.243	0.054	4.189	0.000
CVDF – > EP	0.614	0.052	9.137	0.000
CVD VC* SMF – > EP	−0.023	0.031	2.093	0.000
CVD VC* CVDF – > EP	−0.202	0.039	6.140	0.000

Hypotheses show the influence of independent variables on employee performance. Table 2 shows that the independent variable COVID-19 worry and social media fear have a negative impact on employee job performance with beta value is negative, and *t*-value is 2.093. Social media fear significantly impacts the COVID-19 vaccine, with a beta value of − 0.202 and 6.140. This confirms that the independent variable included in the model has a positive and significant impact on employee performance. Based on the *t*-value independent variable has a positive and significant impact on employee performance at *p* ≤ 0.05.

Table 4 tests the variables by employing the HTMT discrimination validity test, which measures the heterotrait-monotrait discriminant validity. The results presented that the values are very far from 1, not even near to 0.80. The values are confirmed that there is no multicollinearity in selected variables. Table 5 contains the results of regression analysis and presents the values of *R*^2^ and adjusted *R*^2^. The linear regression results have measured the proportion of variations in selected dependent variables toward independent variables with 0.867. The decreasing trend of Adjusted *R*^2^ indicated that the predictions would not improve more according to expectations.

**TABLE 4:** HTMT discriminant validity.

**Sr no.**		**1**	**2**	**3**	**4**
1	Social media fear				
2	COVID-19 worry	0.434			
3	COVID-19 vaccine	0.036	0.024		
4	Employee performance	0.305	0.170	0.064	

**TABLE 5:** Regression analysis.

	***R* ^2^**	**R square adjusted**
COVID-19 vaccine	0.867	0.856

Table 5 shows the moderation effect of the COVID-19 vaccine. First, we assessed the direct relationships before looking at the moderation effects. The results revealed a negative relationship between social media fear and employee performance (β = − 0.023, *p* < 0.00) which gives positive support for H1 of our study. The moderation hypotheses of the COVID-19 vaccine in the path between social media fear and employee performance (H2) are tested using the two-stage continuous moderation analysis (Hair et al., 2017). The moderating effect of SMF^∗^CV – > EP (β = 0.243, *p* < 0.01), indicating the moderating effects are statistically significant at the 0.01 level. This supports H2 of this study. Table 6 presents the summary of Table 3 with understandable and clear manners. The hypothetical approaches are strongly supported by the analytical process.

**TABLE 6:** Hypotheses results.

	**Hypotheses**	
H1	Social media fear significantly influences employee performance.	Supported
H2	COVID-19 worry significantly influences employee performance.	Supported
H3	COVID-19 vaccine moderates the relationship between social media fear and employee performance	Supported
H4	COVID-19 vaccine moderates the relationship between COVID-19 fear and employee performance	Supported

## Discussion

In this study, we used a large sample of social media users; we tested the theory that employees who are more sensitive to emotion contagion would experience more anguish and display symptoms during the COVID-19 pandemic in China. Individuals with heightened fear, or who are more affected by the emotions of others, were more likely to have greater degrees of fear, sorrow, anxiety, and stress, according to COVID-19 results. These details are covered in more detail in the section below.

Although the variations in sensitivity to COVID-19 worry and fear of social media were found to be small, generally, we observed that those with greater vulnerability to contagion were more likely to exhibit worry about the virus and maladaptive reactions (i.e., OCD symptoms). This result is consistent with other research, which showed that those with a greater capacity for emotional contagion tend to have a more pronounced stress reaction to stressful experiences (Trautmann et al., 2018; Sarfraz et al., 2020). Because of the current COVID-19 epidemic, the community as a whole is under stress. Those who are very sensitive to others’ emotional states are likely to be more vulnerable to feelings of worry.

Additionally, we examined the potential that general social media usage and media consumption regarding COVID-19, in particular, might be significant in predicting the degree of anxiety around COVID-19. Additionally, we examined the possibility that the COVID-19 vaccine might serve as a moderator in these interactions. Consumption of media regarding COVID-19 was shown to predict the degree of anxiety associated with COVID-19 significantly, but this connection was not reduced by emotion contagion. One previous study discovered that the degree of emotion contagion moderated the stress response following exposure to a traumatic film. Still, this study was conducted in the laboratory and used a highly controlled stressor, whereas our measure of media exposure on COVID-19 was retrospective and uncontrolled. As a result, it is conceivable that a substantial moderating effect might have developed under more controlled circumstances.

As our further research showed, we discovered that the relationship between COVID-19 worry and fear of social media and employee performance was influenced by the COVID-19 vaccine, making it greater for an employee who was more susceptible to social media fear. The connection between social media fear and employee performance is likely exacerbated because of the increased susceptibility to infection with the virus that causes COVD-19. Despite this warning, it is important to remember that the study was based on the primary analysis of cross-sectional data, meaning no causal conclusions can be drawn. Further studies are also required to examine this in clinical populations.

## Study Limitations

Study limitations must be considered while evaluating current findings. For our study, we first gathered all of our data online using a cross-sectional design, where subjects answered questions at a particular moment in time. Consequently, our findings do not allow us to prove causality. This is because we cannot tell if media exposure raises anxiety or whether those with anxiety gravitate toward news and therefore spend more time on social media. To properly predict the potential effects of a global epidemic, more in-depth research might follow those who exhibit high and low degrees of fear to see how social media is used and the stress levels that develop in reaction to the danger.

Similarly, additional factors that explain the connections between observations are regarded as possible third variables. Unfortunately, we did not measure neuroticism in our survey, which is an interesting area for future study. Moreover, all the data were obtained via self-report surveys, and they may have been influenced by shared method variation since they rely on self-reporting. Future mixed-techniques studies, such as interviews, would diversify the measuring methods used. While the data were restricted to the sample of social media users, they were at last assembled. To validate these results, further research should be conducted in other samples, such as those working in other settings with fear who are especially susceptible to pandemic disease risks (Denis et al., 2020) and those with a higher proportion of male participants. Some of the findings from recent studies have had modest but statistically significant outcomes, and thus they need to be looked at again to see whether they have clinical relevance.

## Conclusion

The study concludes that social media was responsible for spreading COVID-19 fear among people in China. Meanwhile, during this health crisis of the outbreak of COVID-19, trust between citizens and the state had declined, which is why official statements, news, and information provided by the Ministry of Health and government agencies met the needs of people. Therefore, they rely heavily on social media platforms to obtain information about viruses. The nature of social media panic among people depends on gender, age, and level of education. It can be seen that social media has played a crucial role in spreading the fear of the spread of COVID-19 in China and other countries. According to media experts and academics, we believe that we and Chinese comminutes now have an essential role in the future. We must educate media users about good things, reliable information, and a way of thinking critically since younger people are also getting a lot of information through the internet and then spreading it to their family and friends. A university is an ideal place to design courses and symposiums that can help students and teachers figure out how to find, search for and evaluate health information in someone’s case during Epidemics.

The scope of the investigation varies, but those affecting the research process are important. There was a lack of literature on social media about COVID-19, so this study relied on novel research available but was very limited. In addition, conducting research required data, so data collection was another hurdle due to the lockdown period. Because of the data collection, we had difficulty finding participants who wanted to respond and participate.

## Data Availability Statement

The original contributions presented in the study are included in the article/supplementary material, further inquiries can be directed to the corresponding author.

## Author Contributions

All authors listed have made a substantial, direct, and intellectual contribution to the work, and approved it for publication.

## Conflict of Interest

The authors declare that the research was conducted in the absence of any commercial or financial relationships that could be construed as a potential conflict of interest.

## Publisher’s Note

All claims expressed in this article are solely those of the authors and do not necessarily represent those of their affiliated organizations, or those of the publisher, the editors and the reviewers. Any product that may be evaluated in this article, or claim that may be made by its manufacturer, is not guaranteed or endorsed by the publisher.
